# Clinical implications of admission anemia for electroconvulsive therapy planning in adolescent major depressive disorder: identifying vulnerable subgroups with poorer response

**DOI:** 10.3389/fpsyt.2025.1691782

**Published:** 2026-01-15

**Authors:** Dandan Geng, Heyan Xu, Jijia Gou, Yuna Wang, Yujia Chen, Su Hong, Li Kuang

**Affiliations:** 1 Psychiatric Center, First Affiliated Hospital of Chongqing Medical University, Chongqing, China; 2 Mental Health Center, University-Town Hospital of Chongqing Medical University, Chongqing, China

**Keywords:** major depressive disorder, electroconvulsive therapy, anemia, adolescent, treatment outcome

## Abstract

**Background:**

Major depressive disorder (MDD) represents a grave worldwide concern, particularly afflicting the adolescent population. Electroconvulsive therapy (ECT) is widely regarded as a gold-standard intervention for severe forms of MDD, although treatment response varies considerably among individuals. Growing evidence suggests that hematological parameters may influence therapeutic outcomes. This study sought to examine the link between admission anemia and response to ECT treatment.

**Methods:**

We analyzed 381 adolescent MDD patients who underwent ECT, comparing demographic and hematological indicators between responders and non-responders. Subgroup analyses were conducted based on gender and depressive subtypes.

**Results:**

Among the 381 patients treated with ECT, 272 (71.4%) were classified as responders. Non-responders showed significantly lower baseline hemoglobin levels compared to responders (mean ± SD: 119.0 ± 9.7 vs. 128.7 ± 13.1, p < 0.001). Analysis identified a significant link between hemoglobin levels at admission and the percentage improvement on the HAMD-17 (r = 0.231, p < 0.001). Following confounder adjustment in a binary logistic regression model, anemia at admission was correlated with a lower probability of ECT response [OR (95% CI): 4.051 (2.399-6.840), p < 0.001]. Females and patients with psychotic depression were particularly more susceptible to the impact of admission anemia.

**Conclusion:**

Admission anemia is associated with poorer ECT efficacy in adolescent MDD patients. Assessing baseline hemoglobin levels may help optimize ECT treatment planning, especially in female patients and those with psychotic depression.

## 1 Introduction

Major depressive disorder (MDD) constitutes a leading cause of mental health disease in adolescents across the world, with a global prevalence of approximately 6.2% (1). Studies indicate that up to 67% of adolescents with depressive symptoms face a significantly higher risk of developing full-syndrome depression or anxiety disorders in adulthood. Additionally, this age group is more prone to suicidal ideation and self-harm behaviors (2–5). Electroconvulsive therapy (ECT) is widely regarded as one of the most potent therapeutic options for MDD (6, 7), achieving a remission rate of 70%-80% (8). One study reported an 80.9% response rate in adolescent MDD patients treated with ECT (9). However, due to the unclear mechanisms underlying ECT’s therapeutic effects, variability in treatment outcomes, and potential side effects, its widespread use among adolescent MDD patients remains limited. Identifying objective biomarkers predictive of ECT efficacy thus holds significant clinical importance.

In recent years, peripheral blood cell parameters have gained attention in depression research due to their association with inflammation and metabolic status (10–12). Anemia is defined as a condition where the red blood cell (RBC) count or hemoglobin concentration in peripheral blood falls below the normal threshold (13). Hemoglobin, essential for maintaining tissue oxygen metabolism, may influence neuroplasticity by regulating brain energy metabolism, thereby playing a role in various neuropsychiatric conditions, especially Alzheimer’s disease, autism, and depression (14–16). A large cross-sectional study of 44,173 healthy adults revealed a significant and robust association between depression and anemia (17). Furthermore, a 4-year prospective study in older adults demonstrated that depression was independently and positively correlated with both admission anemia and a higher likelihood of anemia at the 4-year follow-up, with this association being more pronounced in MDD (16).

While extensive research over past decades has examined the link between anemia and depression, literature directly investigating the relationship between anemia and the clinical efficacy of ECT remains limited, especially among adolescents. Given that hemoglobin is crucial for cerebral oxygen supply and energy metabolism and may influence neuroplasticity, which is a key mechanism in both depression pathophysiology and ECT treatment (18, 19), we hypothesize that admission anemia may serve as an important modulator of ECT clinical outcomes. This research specifically examines three aspects (1): the correlation between admission anemia and ECT response rates (2); the predictive potential of various blood cell parameters for treatment efficacy; and (3) differential associations between admission anemia and ECT response across demographic and clinical subgroups. To our knowledge, this is the first study specifically designed to investigate the predictive value of admission anemia status for ECT outcomes in adolescent MDD patients. This research aims to address a critical knowledge gap and may provide an easily accessible biomarker for personalized treatment strategies in this challenging patient population.

## 2 Methods

### 2.1 Participants

A retrospective study was conducted on MDD patients admitted to the Department of Psychiatry at the First Affiliated Hospital of Chongqing Medical University between May 2023 and February 2025. Inclusion criteria were (1): patients aged 13–18 years (2); meeting DSM-5 diagnostic criteria for major depressive disorder (3); complete blood count tested within 24 hours of admission; and (4) completion of ECT treatment. Exclusion criteria included (1): inability to complete ECT (2); substance dependence or drug abuse (within the past 6 months) (3); prior ECT treatment (within the past 6 months) (4); history of brain disorders or severe traumatic brain injury (5); recent blood transfusion (within 1 month); and (6) missing laboratory data or assessment scale results. The study protocol was approved by the Ethics Committee of the First Affiliated Hospital of Chongqing Medical University (K2023-676). Written informed consent was obtained from all participants or their legal guardians.

### 2.2 Clinical data acquisition

Clinical characteristics were recorded, such as age, gender, number of ECT sessions, and concomitant medications. Pharmacological treatments were categorized into antidepressants [selective serotonin reuptake inhibitors (SSRIs), serotonin and norepinephrine reuptake inhibitors (SNRIs), mirtazapin, and trazodone], antipsychotics (risperidone, olanzapine, quetiapine, or aripiprazole), and anxiolytics (benzodiazepines and Z-drugs). The baseline fasting blood samples were collected from all patients within 24 hours after admission and prior to the initiation of ECT. The first ECT session was administered within 1–2 days following the blood draw, ensuring a consistent pre-treatment baseline assessment across the entire cohort. Complete blood count was analyzed using Sysmex XN-1500 automated hematology analyzer. Anemia was diagnosed according to WHO criteria: hemoglobin <120 g/L for females and males aged 12–14 years; <120 g/L for females and <130 g/L for males aged 15–18 years (20).

The 17-item Hamilton Depression Rating Scale (HAMD-17) was utilized to evaluate depression severity and treatment response. A positive response to ECT was defined as ≥ 50% reduction in HAMD-17 scores from baseline (21).

### 2.3 ECT procedure

All ECT sessions were delivered via the Thymatron DGx system (Somatics LLC, Lake Bluff, IL, USA), employing brief-pulse stimulus with bitemporal electrode application. A standardized formula (age × 0.7) defined the initial electrical charge administered, with subsequent adjustments based on seizure duration. Induction of anesthesia was achieved with succinylcholine (0.5–1 mg/kg) and propofol (1.5–2 mg/kg). We obtained written informed consent from all patients and their legal guardians prior to treatment, after comprehensively explaining the procedures, risks, and benefits of ECT.

### 2.4 Statistical analysis

Categorical variables were assessed between groups with chi-square, as appropriate, and are expressed as frequencies and percentages. Analysis of continuous variables was conducted using t-tests or Mann-Whitney U tests, reported as mean ± SD or median (IQR). Correlation analyses used Pearson/Spearman tests. We constructed receiver operating characteristic (ROC) curves to assess the ability of blood cell indices to predict ECT outcomes. Logistic regression was performed to calculate odds ratios (OR) with 95% confidence intervals (CI) for the association between anemia and ECT response. Formal testing for interaction effects between admission anemia and categorical covariates, including age, gender, and depressive subtype, was performed by introducing product terms into binary logistic regression models containing the main effects of both variables. The significance of these interaction terms was assessed using the Wald test, and the resulting p-values are reported as p-interaction. To illustrate the association between anemia and ECT response within each subgroup, we subsequently performed stratified analyses and reported the stratum-specific OR and p-values. Statistical significance was established at the p < 0.05 level. Analyses were carried out utilizing IBM-SPSS 23.0 and Prism 10.0.

## 3 Results

### 3.1 Patient demographic characteristics

**Figure 1:**
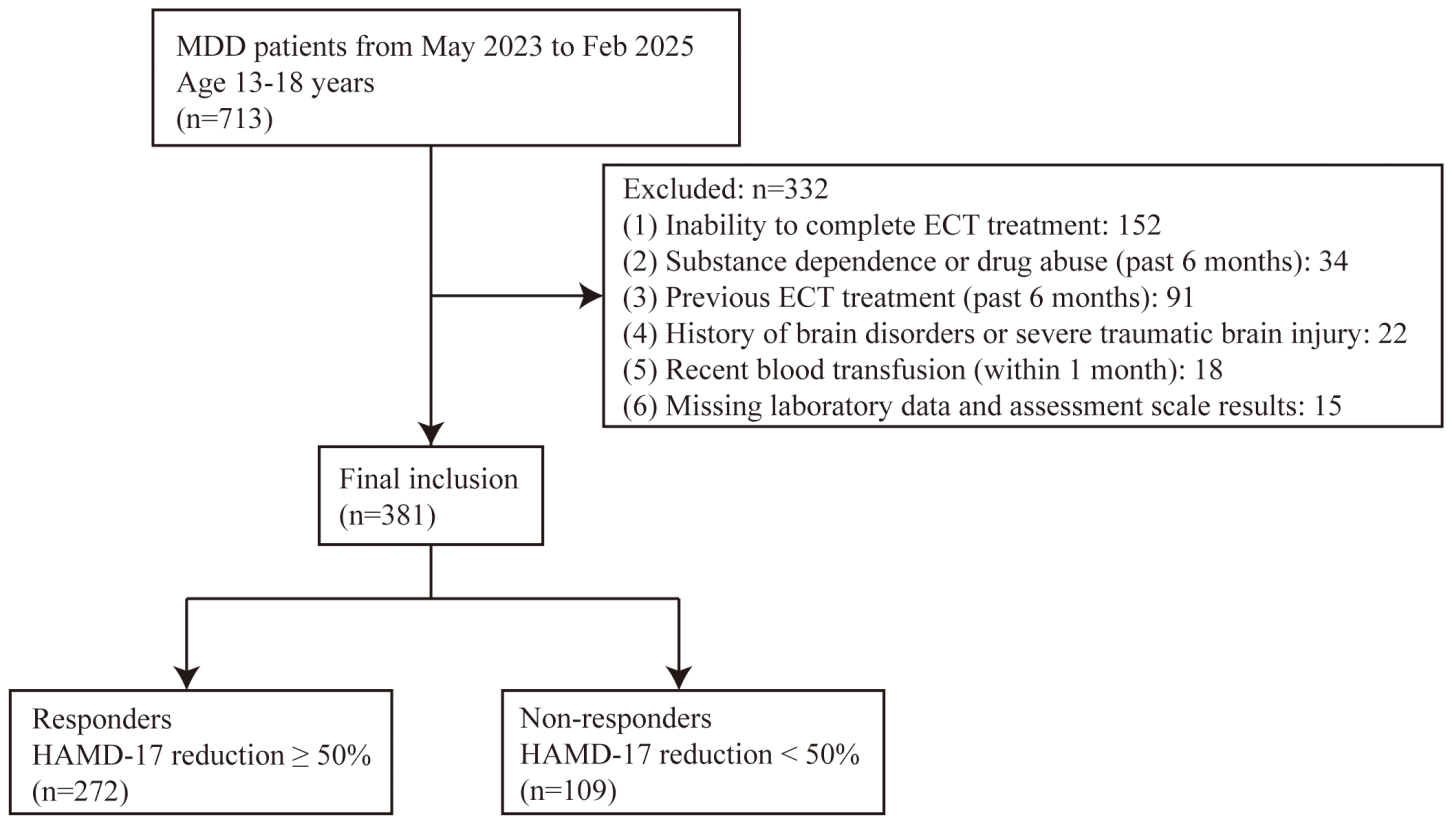
Study population recruitment flow chart. MDD, major depressive disorder; ECT, electroconvulsive therapy; HAMD-17, Hamilton depression rating scale, 17-item version.

From May 2023 to February 2025, we recruited a cohort of 713 adolescents diagnosed with MDD. Following the screening process, 332 patients were excluded due to failure to meet one or more of the following criteria: 152 cases due to inability to complete ECT treatment (comprising 65 patients who withdrew consent due to concerns about anesthesia or cognitive side effects after initial sessions; 40 patients who discontinued due to significant adverse effects such as severe headache or prolonged confusion; 32 patients discharged against medical advice for non-medical reasons including financial constraints or family decisions; and 15 patients who developed intercurrent physical illnesses unrelated to hematological issues requiring ECT cessation), 34 cases with substance dependence or drug abuse (within the past 6 months), and 91 cases with prior ECT treatment (within the past 6 months). Ultimately, 381 adolescent MDD patients were enrolled in the study, with 272 responders (71.4%) and 109 non-responders (28.6%) (**Figure 1**). The study cohort comprised 245 female participants (64.3%), with mean values of 14.7 ± 1.3 years for age and 20.7 ± 3.7 kg/m² for BMI, respectively (**Table 1**).

**Table 1:** Baseline characteristics of the analyzed participants.

Variables	Total (n = 381)	Responders (n = 272)	Non-Responders (n = 109)	t/U/χ2	p-value
Age, year (mean ± SD)	14.7 ± 1.3	14.5 ± 1.3	14.7 ± 1.2	-1.421	0.157
Female, n (%)	245 (64.3%)	172 (63.2%)	73 (67.0%)	0.473	0.491
BMI, kg/m^2^ (mean ± SD)	20.7 ± 3.7	21.1± 3.7	20.7 ± 3.7	0.953	0.342
Smoking, n (%)	39 (10.2%)	30 (11.0%)	9 (8.3%)	0.651	0.420
Drinking, n (%)	38 (10.0%)	26 (9.6%)	12 (11.0%)	0.182	0.669
Psychotic depression, n (%)	86 (22.6%)	54 (19.9%)	32 (29.4%)	4.022	0.045*
Medications					
SSRI, n (%)	274 (71.9%)	188 (69.1%)	86 (78.9%)	3.686	0.055
SNRI, n (%)	72 (18.9%)	48 (17.6%)	24 (22.0%)	0.970	0.325
Mirtazapin, n (%)	28 (7.3%)	17 (6.3%)	11 (10.1%)	1.687	0.194
Trazodone, n (%)	36 (9.4%)	23 (8.5%)	13 (11.9%)	1.096	0.295
Antipsychotics, n (%)	305 (80.1%)	220 (80.9%)	85 (78.0%)	0.410	0.522
Benzodiazepine, n (%)	185 (48.6%)	128 (47.1%)	57 (52.3%)	0.854	0.356
Z-drug hypnotics, n (%)	134 (35.2%)	98 (36.0%)	36 (33.0%)	0.308	0.579
ECT sessions, (median, IQR)	7.0 (6.0-8.0)	7.0 (6.0-8.0)	7.0 (6.0-8.0)	13711.000	0.402
Adverse effects of ECT					
Slowness of thought, n (%)	23 (6.0%)	19 (7.0%)	4 (3.7%)	1.508	0.219
Memory impairment, n (%)	20 (5.2%)	15 (5.5%)	5 (4.6%)	0.135	0.714
Headache, n (%)	19 (5.0%)	10 (3.7%)	9 (8.3%)	3.446	0.063
Emesis, n (%)	5 (1.3%)	4 (1.5%)	1(0.9%)	0.184	0.668
Haziness, n (%)	7 (1.8%)	4 (1.5%)	3 (2.8%)	0.184	0.400
Baseline HAMD-17, (median, IQR)	33.0 (29.0-38.0)	33.0 (28.0-38.0)	33.0 (29.0-40.0)	13444.500	0.155
Post-ECT HAMD-17, (median, IQR)	12.0 (9.0-18.0)	10.0 (7.0-13.0)	22.0 (17.0-24.0)	1768.000	< 0.001*
WBC, 10*9/L (mean ± SD)	6.2 ± 1.6	5.9 ± 1.4	6.2 ± 1.6	−1.712	0.043*
RBC, 10*12/L (mean ± SD)	4.5 ± 0.3	4.5 ± 0.3	4.4 ± 0.3	2.545	0.012*
Hemoglobin, g/L (mean ± SD)	119.0 ± 9.7	128.7 ± 13.1	119.0 ± 9.7	7.997	< 0.001*
Anemia, n (%)	125 (32.8%)	64 (23.5%)	61 (56.0%)	37.134	< 0.001*
Hematocrit, % (mean ± SD)	39.9 ± 6.0	40.1 ± 5.6	39.9 ± 6.0	0.457	0.649
MPV, fL (mean ± SD)	10.5 ± 1.1	10.5 ± 1.0	10.5 ± 1.1	-0.065	0.948
Platelets, 10*9/L (mean ± SD)	236.8 ± 59.6	241.2 ± 57.1	232.3 ± 65.7	1.237	0.218
Neutrophils, 10*9/L (median, IQR)	2.7 (2.2-3.6)	2.8 (2.2-3.6)	2.6 (2.2-3.6)	14558.500	0.785
Lymphocyte, 10*9/L (median, IQR)	2.0 (1.7-2.6)	2.1 (1.7-2.6)	1.9 (1.7-2.7)	14275.500	0.572
Monocyte, 10*9/L (mean ± SD)	0.4 ± 0.1	0.5 ± 0.1	0.4 ± 0.1	1.151	0.251

BMI, body mass index; SSRI, selective serotonin reuptake inhibitor; SNRI, serotonin-norepinephrine reuptake inhibitor; ECT, electroconvulsive therapy; HAMD-17, Hamilton depression rating scale, 17-item version; WBC, white blood cell; RBC, red blood cell; MPV, mean platelet volume; *p values considered statistically significant; *p < 0.05.

### 3.2 Differences in clinical characteristics between ECT responders and non-responders

**Figure 2:**
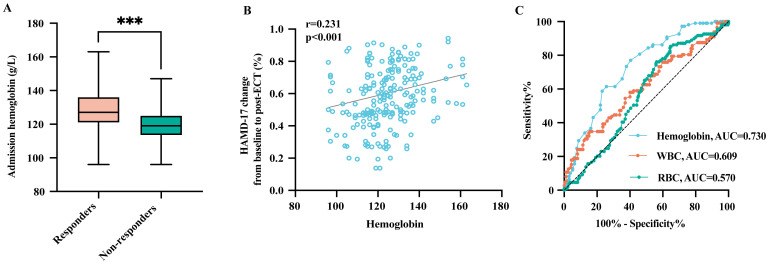
The association between baseline hemoglobin levels with ECT treatment outcomes in adolescent MDD patients. **(A)** Baseline hemoglobin levels in ECT responders and non-responders; **(B)** Correlation between baseline hemoglobin levels and percentage change in HAMD-17 scores; **(C)** Receiver operating characteristic analysis of blood cell parameters for predicting ECT response. MDD, major depressive disorder; ECT, electroconvulsive therapy; AUC, area under the curve; WBC, white blood cell; RBC, red blood cell. ***p < 0.001.

Comparative analysis of clinical characteristics between response groups is summarized in **Table 1**. The baseline hemoglobin level was significantly lower in non-responders than in responders (mean ± SD: 119.0 ± 9.7 vs. 128.7 ± 13.1, p < 0.001) (**Table 1**, **Figure 2A**). Anemia was present in 56% of non-responders, compared to only 23.5% in responders (p < 0.001). Additionally, non-responders had lower RBC counts (mean ± SD: 4.4 ± 0.3 vs. 4.5 ± 0.3, p = 0.012) but higher WBC counts (mean ± SD: 6.2 ± 1.6 vs. 5.9 ± 1.4, p = 0.043). Notably, psychotic depression was more prevalent in non-responders (29.4% vs. 19.9%, p = 0.045). However, analysis revealed no significant differences in age, gender, BMI, antidepressants, antipsychotics, or other factors (p > 0.05) (**Table 1**).

Correlation analysis revealed that baseline hemoglobin levels were positively associated with the percentage improvement on the HAMD-17 (r = 0.231, p < 0.001) (**Figure 2B**). Furthermore, ROC curve analysis showed that hemoglobin had greater predictive power for ECT response in adolescent MDD patients (AUC = 0.730, sensitivity = 77.1%, specificity = 59.6%) compared to other blood cell markers, such as WBC [AUC = 0.609, 95% CI (0.545–0.673), p < 0.001] and RBC [AUC = 0.570, 95% CI (0.510–0.631), p = 0.030] (**Figure 2C**).

### 3.3 Baseline characteristics of anemia and non-anemia stratified by admission hemoglobin levels

All adolescent MDD patients were divided into anemia (32.8%) and non-anemia (67.2%) groups based on hemoglobin levels at admission. Compared to the non-anemia patients, patients with anemia had a higher likelihood of experiencing adverse effects from ECT, including increased risks of memory impairment (8.8% vs. 3.5%, p = 0.030), headache (9.6% vs. 2.7%, p = 0.004), and haziness (4.0% vs. 0.8%, p = 0.028). Additionally, the anemia group exhibited higher post-ECT HAMD-17 scores [median (IQR): 15.0 (10.0–21.0) vs. 11.0 (8.0–16.0), p < 0.001] and a lower proportion of ECT responders (51.2% vs. 81.3%, p < 0.001) (**Table 2**). These findings suggest that admission anemia is significantly associated with both ECT-related side effects and treatment efficacy.

**Table 2:** Baseline profiles of MDD patients categorized by anemia status at admission.

Variables	Anemia (n = 125)	Non-anemia (n = 256)	t/U/χ2	p-value
Age, year (mean ± SD)	14.5 ± 1.4	14.6 ± 1.4	0.047	0.963
Female, n (%)	85 (68.0%)	160 (62.5%)	1.107	0.293
BMI, kg/m^2^ (mean ± SD)	20.5 ± 3.8	21.1 ± 3.6	1.479	0.141
Smoking, n (%)	10 (8.0%)	29 (11.3%)	1.012	0.314
Drinking, n (%)	11 (8.8%)	27 (10.5%)	0.285	0.593
Psychotic depression, n (%)	27 (21.6%)	59 (23.0%)	0.101	0.751
Medications				
SSRI, n (%)	89 (71.2%)	185 (72.3%)	0.047	0.828
SNRI, n (%)	30 (24.0%)	42 (16.4%)	3.160	0.075
Mirtazapin, n (%)	12 (9.6%)	16 (6.3%)	1.384	0.239
Trazodone, n (%)	15 (12.0%)	21 (8.2%)	1.415	0.234
Antipsychotics, n (%)	100 (80.0%)	205 (80.1%)	< 0.001	0.986
Benzodiazepine, n (%)	67 (53.6%)	118 (46.1%)	1.894	0.169
Z-drug hypnotics, n (%)	51 (40.8%)	83 (32.4%)	2.586	0.108
ECT sessions, (median, IQR)	7.0 (6.0-8.0)	7.0 (6.0-8.0)	14352.500	0.191
Adverse effects of ECT				
Slowness of thought, n (%)	16 (6.3%)	7 (5.6%)	0.063	0.802
Memory impairment, n (%)	11 (8.8%)	9 (3.5%)	4.715	0.030*
Headache, n (%)	12 (9.6%)	7 (2.7%)	8.356	0.004*
Emesis, n (%)	2 (1.6%)	3 (1.2%)	0.119	0.730
Haziness, n (%)	5 (4.0%)	2 (0.8%)	4.825	0.028*
Baseline HAMD-17, (median, IQR)	34.0 (28.0-40.0)	33.0 (29.0-38.0)	15399.500	0.551
Post-ECT HAMD-17, (median, IQR)	15.0 (10.0-21.0)	11.0 (8.0-16.0)	11882.000	< 0.001*
Responders, n (%)	64 (51.2%)	208 (81.3%)	37.134	< 0.001*

BMI, body mass index; SSRI, selective serotonin reuptake inhibitor; SNRI, serotonin-norepinephrine reuptake inhibitor; ECT, electroconvulsive therapy; HAMD-17, Hamilton depression rating scale, 17-item version; *p values considered statistically significant; *p < 0.05.

### 3.4 Predictors of ECT response

In binary logistic regression analysis after adjusting for potential confounders (including age, gender, BMI, antipsychotics, antidepressants, anxiolytics and variables with p < 0.1 in univariate analysis), we found that anemia [OR (95% CI): 4.051 (2.399-6.840), p < 0.001] significantly reduced the likelihood of ECT response (**Table 3**). This indicates that anemia is independently associated with ECT treatment outcomes.

**Table 3:** Binary logistic regression analysis of admission anemia and ECT response.

Variables	Model 1			Model 2		
	B	OR (95% CI)	*p*-value	B	OR (95% CI)	*p*-value
Anemia	1.413	4.107 (2.555-6.601)	< 0.001*	1.399	4.051 (2.399-6.840)	< 0.001*
Age	0.140	1.150(0.963-1.372)	0.122	0.148	1.159 (0.953-1.411)	0.140
Female	0.096	1.100(0.671-1.806)	0.705	0.095	1.100 (0.654-1.852)	0.720
BMI	-0.022	0.978(0.917-1.044)	0.507	-0.019	0.981 (0.914-1.053)	0.593
WBC				0.100	1.105 (0.958-1.275)	0.170
RBC				-0.711	0.491 (0.217-1.109)	0.087
Antidepressants				0.846	2.33 (0.963-5.640)	0.061
Antipsychotics				-0.057	0.945 (0.505-1.769)	0.860
Anxiolytics				-0.378	0.685 (0.401-1.172)	0.167
Psychotic depression				0.518	1.679 (0.915-3.081)	0.094
Headache				0.677	1.968 (0.706-5.485)	0.196

BMI, body mass index; SSRI, selective serotonin reuptake inhibitor; WBC, white blood cell; RBC, red blood cell; B, unstandardized coefficient; OR, odds ratio; CI, confidence interval.

Model 1: adjusted for age, gender, and BMI.

Model 2: adjusted for variables in Model 1 plus SSRI, WBC, RBC, antidepressants, antipsychotics, anxiolytics, psychotic depression, and headache.

*p values considered statistically significant; *p < 0.05.

### 3.5 Subgroup differences in the association between anemia and ECT response

We investigated potential interactions between admission anemia and various demographic and clinical factors. As shown in **Table 4**, after stratifying by age, gender, and depressive subtype, a significant interaction effect was observed between anemia and gender [female vs. male, OR (95% CI): 29.292 (11.644–73.687) vs. 0.513 (0.180–1.461), p for interaction < 0.001]. These findings suggest gender-specific effects - anemia was independently associated with poor ECT response in female patients but not in males. Additionally, an interaction effect was also found in patients with psychotic depression [none vs. yes, OR (95% CI): 3.388 (1.868–6.143) vs. 21.560 (6.588–70.552), p for interaction = 0.027], indicating that individuals with psychotic depression may be more sensitive to anemia at admission (**Table 4**).

**Table 4:** Subgroup analysis indicating the association between adm. anemia and ECT response.

Subgroup variables	ECT response	OR (95% CI)	p-value	p-_interaction_
Age, years				
< 15 (n = 230)	163 (70.9%)	4.433 (2.207-8.905)	< 0.001	0.864
≥ 15 (n = 151)	109 (72.2%)	8.629 (3.238-22.991)	< 0.001	
Gender				
Male (n = 136)	100 (73.5%)	0.513 (0.180-1.461)	0.221	< 0.001*
Female (n = 245)	172 (70.2%)	29.292 (11.644-73.687)	< 0.001	
Psychotic depression				
None (n = 295)	218 (73.9%)	3.388 (1.868-6.143)	< 0.001	0.027*
Yes (n = 86)	54 (62.8%)	21.560 (6.588-70.552)	< 0.001	

Odds ratio (OR) for admission anemia and ECT response were stratified by some characteristics, such as age, gender, and psychotic depression. OR for incidences of ECT non-response were adjusted for variables of model 2 in **Table 3**.

*p values considered statistically significant; *p <0.05.

## 4 Discussion

This study provides the systematic evaluation of the relationship between complete blood cell parameters and ECT efficacy in adolescents, aiming to identify easily accessible biomarkers for predicting ECT response and optimizing treatment selection for adolescent MDD patients to achieve personalized intervention. Our results demonstrate a significant association between anemia at admission and poor ECT response, with non-responders showing lower hemoglobin levels, reduced RBC counts, and elevated WBC counts. Hemoglobin emerged as a superior predictor of ECT efficacy compared to other blood cell parameters. Notably, patients with anemia experienced a higher incidence of post-ECT adverse effects, including memory impairment, headaches, and haziness. Furthermore, the predictive value of anemia appears to vary across subgroups, showing particularly strong associations in female patients and those with psychotic depression subtypes.

Anemia is defined as a condition characterized by hemoglobin concentration and/or RBC count below normal levels, insufficient to meet an individual’s physiological needs (22). A large cross-sectional study of 11,876 Japanese participants revealed a significant association between self-reported history of depression and history of iron-deficiency anemia (23), with evidence suggesting this relationship may be bidirectional (24, 25). Research involving 1,156 elderly participants demonstrated that the risk of anemia increases with depression severity (26). Additionally, anemia may contribute to the development of depression. In another study involving 223 patients with acute coronary syndrome, the presence of anemia at admission was found to increase the risk of developing depression three weeks after hospitalization (27), consistent with our current findings. In adolescent MDD patients, we observed that anemia independently correlated with reduced response rates to ECT treatment.

How might admission anemia affect ECT response? One possible explanation involves a direct cerebral mechanism related to impaired oxygen delivery. Anemia compromises cerebral oxygen metabolism, which is crucial for neuronal function (26, 28, 29). This is particularly critical for brain regions with high metabolic demands and established roles in MDD, such as the prefrontal cortex (responsible for executive function and emotion regulation), the hippocampus (critical for memory and stress response), and the temporal lobe (30–32). We hypothesize that a brain already experiencing anemic hypoxia may have a diminished capacity to support the substantial metabolic demands required for ECT-induced neuroplasticity. This could potentially weaken the treatment response and increase susceptibility to ECT-related adverse effects, particularly cognitive side effects such as memory impairment. Additionally, systemic effects of anemia, including fatigue and reduced physiological resilience (33–35), may further lower the tolerance threshold for somatic side effects like headaches. This aligns with our study findings, which showed a higher incidence of post-ECT memory loss and headaches reported in the anemia group. Beyond this direct cerebral effect, inflammation may constitute a shared pathological basis linking anemia to treatment resistance. Chronic inflammation is a recognized factor contributing to both the pathophysiology of depression and anemia of inflammation (36, 37). Proinflammatory cytokines can disrupt neuronal activity, alter neuronal excitability, and elevate seizure thresholds (38, 39). This may necessitate higher stimulation intensities to achieve adequate seizure outcomes. Furthermore, inflammation impairs neurotrophic signaling and suppresses neurogenesis (40, 41), thereby interfering with neural plasticity changes that underlie sustained antidepressant effects. Our finding of higher baseline WBC counts in ECT non-responders provides preliminary clinical support for the role of an inflammatory state in mediating poor outcomes. Therefore, from a clinical perspective, these insights suggest that proactively identifying and correcting anemia before ECT could be a strategic approach to potentially enhance treatment efficacy while reducing treatment-related morbidity.

Our study revealed an intriguing finding regarding the differential predictive value of anemia across subgroups. Female patients demonstrated greater sensitivity to anemia, which correlated with poorer ECT response, while no significant association was observed in males. This sexual dimorphism may be explained by sex hormones’ known influence on neuroinflammation and neuroendocrine systems (42–44), with hormonal fluctuations potentially amplifying depression susceptibility in young women (45–48), thereby accentuating anemia’s impact on ECT outcomes. Besides, the predictive power of anemia was particularly pronounced in patients with psychotic depression. Existing research indicates significantly reduced striatal dopamine transporter availability in psychotic depression (49, 50), suggesting impaired dopaminergic neurotransmission. Anemia, particularly iron-deficiency anemia, may exacerbate this pathological process, as iron is a critical cofactor for tyrosine hydroxylase, the rate-limiting enzyme in dopamine biosynthesis (51, 52). This dual pathology could worsen psychotic symptoms and diminish ECT efficacy.

Several limitations warrant consideration: First, we only assessed hemoglobin levels at admission without tracking dynamic changes during hospitalization, which might provide more accurate correlation data. Second, the absence of vitamin and mineral measurements prevented determination of anemia etiology. Third, although we adjusted for several confounders, unexamined factors like diet, iron metabolism, inflammatory markers (e.g., CRP, interleukins), and socioeconomic status may influence outcomes. Fourth, the retrospective design precludes causal inferences between anemia and ECT response. Future prospective cohort studies are needed to directly investigate whether correcting anemia prior to ECT initiation improves clinical outcomes and reduces treatment-related side effects. Furthermore, the clinical utility of hemoglobin as a standalone predictor may be limited by its moderate specificity (59.6%). Future research should focus on developing multi-parameter models that integrate hemoglobin with other biomarkers, such as inflammatory markers, and clinical features. Exploring optimal diagnostic thresholds to balance sensitivity and specificity is also warranted. Finally, moving beyond traditional diagnostic boundaries, subsequent investigations should prioritize developing sex-specific prediction models to enable refined clinical risk assessment. This study provides proof-of-concept for the role of hematological factors in predicting ECT response, laying the foundation for developing more accurate predictive algorithms.

## 5 Conclusion

Our study demonstrates a significant association between admission anemia and poorer ECT response in adolescent MDD patients. Baseline hemoglobin screening may serve as a practical biomarker to identify high-risk individuals, enabling optimized treatment strategies and enhanced management of post-ECT adverse effects. These findings highlight the need for personalized interventions, particularly in female patients and those with psychotic depression, where anemia correction prior to ECT may improve clinical outcomes.

## Data Availability

The raw data supporting the conclusions of this article will be made available by the authors, without undue reservation.

## References

[1] Recchia F Bernal JDK Fong DY Wong SHS Chung PK Chan DKC . Physical activity interventions to alleviate depressive symptoms in children and adolescents: A systematic review and meta-analysis. JAMA Pediatr. (2023) 177:132–40. doi: 10.1001/jamapediatrics.2022.5090, PMID: 36595284 PMC9857695

[2] Nguyen TD Harder A Xiong Y Kowalec K Hägg S Cai N . Genetic heterogeneity and subtypes of major depression. Mol Psychiatry. (2022) 27:1667–75. doi: 10.1038/s41380-021-01413-6, PMID: 34997191 PMC9106834

[3] Thapar A Eyre O Patel V Brent D . Depression in young people. Lancet. (2022) 400:617–31. doi: 10.1016/S0140-6736(22)01012-1, PMID: 35940184

[4] Avenevoli S Swendsen J He JP Burstein M Merikangas KR . Major depression in the national comorbidity survey-adolescent supplement: prevalence, correlates, and treatment. J Am Acad Child Adolesc Psychiatry. (2015) 54:37–44.e2. doi: 10.1016/j.jaac.2014.10.010, PMID: 25524788 PMC4408277

[5] Hsieh MH . Electroconvulsive therapy for treatment-resistant depression. Prog Brain Res. (2023) 281:69–90. doi: 10.1016/bs.pbr.2023.01.004, PMID: 37806717

[6] Leaver AM Espinoza R Wade B Narr KL . Parsing the network mechanisms of electroconvulsive therapy. Biol Psychiatry. (2022) 92:193–203. doi: 10.1016/j.biopsych.2021.11.016, PMID: 35120710 PMC9196257

[7] Zhong Y Li J Li H Li M Lyu Y Cui M . The neuroimaging role of modified electroconvulsive therapy in the major depressive disorder: effectiveness in first-episode antipsychotic-naive major depressive disorder patients. Depress Anxiety. (2024) 2024:9211145. doi: 10.1155/2024/9211145, PMID: 40226641 PMC11919022

[8] Milev RV Giacobbe P Kennedy SH Blumberger DM Daskalakis ZJ Downar J . Canadian network for mood and anxiety treatments (CANMAT) 2016 clinical guidelines for the management of adults with major depressive disorder: section 4. Neurostimulation treatments. Can J Psychiatry. (2016) 61:561–75. doi: 10.1177/0706743716660033, PMID: 27486154 PMC4994792

[9] Li H Hou L Wang D Wu Q Li H He W . Response rate and safety of antidepressants combined with electroconvulsive therapy in adolescent depression: Real-world clinical application. J Affect Disord. (2023) 339:98–103. doi: 10.1016/j.jad.2023.06.052, PMID: 37390926

[10] Ryan KM Lynch M McLoughlin DM . Blood cell ratios in mood and cognitive outcomes following electroconvulsive therapy. J Psychiatr Res. (2022) 156:729–36. doi: 10.1016/j.jpsychires.2022.11.016, PMID: 36413934

[11] Wang L Wang M Liu X Tian J Zhang L Li Y . The association between uric acid to high-density cholesterol ratio and depression: A population-based cross-sectional study. J Affect Disord. (2025) 379:502–9. doi: 10.1016/j.jad.2025.03.023, PMID: 40054537

[12] Liu P-L Zhang Y Li J Du J Yang N Dong Q-L . The association between obesity and depressive symptoms: mediation by C-reactive protein and neutrophil-to-lymphocyte ratio. AP. (2025) 26:45975. doi: 10.31083/AP45975, PMID: 40926826 PMC12416059

[13] Kassebaum NJ . The global burden of anemia. Hematol Oncol Clin North Am. (2016) 30:247–308. doi: 10.1016/j.hoc.2015.11.002, PMID: 27040955

[14] Qiang YX Deng YT Zhang YR Wang HF Zhang W Dong Q . Associations of blood cell indices and anemia with risk of incident dementia: A prospective cohort study of 313,448 participants. Alzheimers Dement. (2023) 19:3965–76. doi: 10.1002/alz.13088, PMID: 37102212

[15] Gardener H Spiegelman D Buka SL . Perinatal and neonatal risk factors for autism: a comprehensive meta-analysis. Pediatrics. (2011) 128:344–55. doi: 10.1542/peds.2010-1036, PMID: 21746727 PMC3387855

[16] Liu C Zhou R Peng X Zhu T Wei W Hao X . Relationship between depressive symptoms and anemia among the middle-aged and elderly: a cohort study over 4-year period. BMC Psychiatry. (2023) 23:572. doi: 10.1186/s12888-023-05047-6, PMID: 37553590 PMC10408197

[17] Vulser H Wiernik E Hoertel N Thomas F Pannier B Czernichow S . Association between depression and anemia in otherwise healthy adults. Acta Psychiatr Scand. (2016) 134:150–60. doi: 10.1111/acps.12595, PMID: 27238642

[18] Rose D Fleischmann P Wykes T Leese M Bindman J . Patients’ perspectives on electroconvulsive therapy: systematic review. Bmj. (2003) 326:1363. doi: 10.1136/bmj.326.7403.1363, PMID: 12816822 PMC162130

[19] Anderson IM McAllister-Williams RH Downey D Elliott R Loo C . Cognitive function after electroconvulsive therapy for depression: relationship to clinical response. Psychol Med. (2021) 51:1647–56. doi: 10.1017/S0033291720000379, PMID: 32102725 PMC8327625

[20] Pasricha SR Rogers L Branca F Garcia-Casal MN . Measuring haemoglobin concentration to define anaemia: WHO guidelines. Lancet. (2024) 403:1963–6. doi: 10.1016/S0140-6736(24)00502-6, PMID: 38493792

[21] Vos CF Birkenhäger TK Nolen WA van den Broek WW Coenen MJH Ter Hark SE . Association of the neutrophil to lymphocyte ratio and white blood cell count with response to pharmacotherapy in unipolar psychotic depression: An exploratory analysis. Brain Behav Immun Health. (2021) 16:100319. doi: 10.1016/j.bbih.2021.100319, PMID: 34423321 PMC7611545

[22] Stevens GA Paciorek CJ Flores-Urrutia MC Borghi E Namaste S Wirth JP . National, regional, and global estimates of anaemia by severity in women and children for 2000-19: a pooled analysis of population-representative data. Lancet Glob Health. (2022) 10:e627–e39. doi: 10.1016/S2214-109X(22)00084-5, PMID: 35427520 PMC9023869

[23] Hidese S Saito K Asano S Kunugi H . Association between iron-deficiency anemia and depression: A web-based Japanese investigation. Psychiatry Clin Neurosci. (2018) 72:513–21. doi: 10.1111/pcn.12656, PMID: 29603506

[24] Park GN Kim JO Oh JW Lee S . Association between anemia and depression: The 2014, 2016, and 2018 Korea National Health and Nutrition Examination Survey. J Affect Disord. (2022) 312:86–91. doi: 10.1016/j.jad.2022.06.015, PMID: 35750091

[25] Young LM Pipingas A White DJ Gauci S Scholey A . A systematic review and meta-analysis of B vitamin supplementation on depressive symptoms, anxiety, and stress: effects on healthy and ‘At-risk’ Individuals. Nutrients. (2019) 11:2232. doi: 10.3390/nu11092232, PMID: 31527485 PMC6770181

[26] Onder G Penninx BW Cesari M Bandinelli S Lauretani F Bartali B . Anemia is associated with depression in older adults: results from the InCHIANTI study. J Gerontol A Biol Sci Med Sci. (2005) 60:1168–72. doi: 10.1093/gerona/60.9.1168, PMID: 16183958

[27] Steptoe A Wikman A Molloy GJ Kaski JC . Anaemia and the development of depressive symptoms following acute coronary syndrome: longitudinal clinical observational study. BMJ Open. (2012) 2:e000551. doi: 10.1136/bmjopen-2011-000551, PMID: 22307099 PMC3274712

[28] Hare GM . Anaemia and the brain. Curr Opin Anaesthesiol. (2004) 17:363–9. doi: 10.1097/00001503-200410000-00003, PMID: 17023891

[29] Kamata T Hishida A Takita T Sawada K Ikegaya N Maruyama Y . Morphologic abnormalities in the brain of chronically hemodialyzed patients without cerebrovascular disease. Am J Nephrol. (2000) 20:27–31. doi: 10.1159/000013551, PMID: 10644864

[30] Ho TC . Stress and neurodevelopment in adolescent depression. Biol Psychiatry. (2019) 86:e33–e5. doi: 10.1016/j.biopsych.2019.09.012, PMID: 31648684 PMC7594880

[31] Cao P Chen C Liu A Shan Q Zhu X Jia C . Early-life inflammation promotes depressive symptoms in adolescence via microglial engulfment of dendritic spines. Neuron. (2021) 109:2573–89.e9. doi: 10.1016/j.neuron.2021.06.012, PMID: 34233151

[32] García-Cabrerizo R Ledesma-Corvi S Bis-Humbert C García-Fuster MJ . Sex differences in the antidepressant-like potential of repeated electroconvulsive seizures in adolescent and adult rats: Regulation of the early stages of hippocampal neurogenesis. Eur Neuropsychopharmacol. (2020) 41:132–45. doi: 10.1016/j.euroneuro.2020.10.008, PMID: 33160794

[33] Penninx BW Pahor M Cesari M Corsi AM Woodman RC Bandinelli S . Anemia is associated with disability and decreased physical performance and muscle strength in the elderly. J Am Geriatr Soc. (2004) 52:719–24. doi: 10.1111/j.1532-5415.2004.52208.x, PMID: 15086651

[34] Cella D Kallich J McDermott A Xu X . The longitudinal relationship of hemoglobin, fatigue and quality of life in anemic cancer patients: results from five randomized clinical trials. Ann Oncol. (2004) 15:979–86. doi: 10.1093/annonc/mdh235, PMID: 15151958

[35] Kallich JD Tchekmedyian NS Damiano AM Shi J Black JT Erder MH . Psychological outcomes associated with anemia-related fatigue in cancer patients. Oncol (Williston Park). (2002) 16:117–24., PMID: 12380961

[36] Irwin MR Miller AH . Depressive disorders and immunity: 20 years of progress and discovery. Brain Behav Immun. (2007) 21:374–83. doi: 10.1016/j.bbi.2007.01.010, PMID: 17360153

[37] Esposito K Marfella R Ciotola M Di Palo C Giugliano F Giugliano G . Effect of a mediterranean-style diet on endothelial dysfunction and markers of vascular inflammation in the metabolic syndrome: a randomized trial. Jama. (2004) 292:1440–6. doi: 10.1001/jama.292.12.1440, PMID: 15383514

[38] Catenoix H Grabon W Rheims S Vukusic S Marignier R . Multiple sclerosis and epilepsy. Rev Neurol (Paris). (2025) 181:391–6. doi: 10.1016/j.neurol.2025.03.008, PMID: 40180801

[39] Vezzani A Viviani B . Neuromodulatory properties of inflammatory cytokines and their impact on neuronal excitability. Neuropharmacology. (2015) 96:70–82. doi: 10.1016/j.neuropharm.2014.10.027, PMID: 25445483

[40] Troubat R Barone P Leman S Desmidt T Cressant A Atanasova B . Neuroinflammation and depression: A review. Eur J Neurosci. (2021) 53:151–71. doi: 10.1111/ejn.14720, PMID: 32150310

[41] Rose-John S Jenkins BJ Garbers C Moll JM Scheller J . Targeting IL-6 trans-signalling: past, present and future prospects. Nat Rev Immunol. (2023) 23:666–81. doi: 10.1038/s41577-023-00856-y, PMID: 37069261 PMC10108826

[42] Freeman EW Sammel MD Lin H Nelson DB . Associations of hormones and menopausal status with depressed mood in women with no history of depression. Arch Gen Psychiatry. (2006) 63:375–82. doi: 10.1001/archpsyc.63.4.375, PMID: 16585466

[43] Frokjaer VG Pinborg A Holst KK Overgaard A Henningsson S Heede M . Role of serotonin transporter changes in depressive responses to sex-steroid hormone manipulation: A positron emission tomography study. Biol Psychiatry. (2015) 78:534–43. doi: 10.1016/j.biopsych.2015.04.015, PMID: 26004162

[44] Klein SL . The effects of hormones on sex differences in infection: from genes to behavior. Neurosci Biobehav Rev. (2000) 24:627–38. doi: 10.1016/S0149-7634(00)00027-0, PMID: 10940438

[45] Faravelli C Alessandra Scarpato M Castellini G Lo Sauro C . Gender differences in depression and anxiety: the role of age. Psychiatry Res. (2013) 210:1301–3. doi: 10.1016/j.psychres.2013.09.027, PMID: 24135551

[46] Kwong ASF López-López JA Hammerton G Manley D Timpson NJ Leckie G . Genetic and environmental risk factors associated with trajectories of depression symptoms from adolescence to young adulthood. JAMA Netw Open. (2019) 2:e196587. doi: 10.1001/jamanetworkopen.2019.6587, PMID: 31251383 PMC6604106

[47] Bekhbat M Neigh GN . Sex differences in the neuro-immune consequences of stress: Focus on depression and anxiety. Brain Behav Immun. (2018) 67:1–12. doi: 10.1016/j.bbi.2017.02.006, PMID: 28216088 PMC5559342

[48] Kuehner C . Why is depression more common among women than among men? Lancet Psychiatry. (2017) 4:146–58. doi: 10.1016/S2215-0366(16)30263-2, PMID: 27856392

[49] Tamura T Sugihara G Okita K Mukai Y Matsuda H Shiwaku H . Dopamine dysfunction in depression: application of texture analysis to dopamine transporter single-photon emission computed tomography imaging. Transl Psychiatry. (2022) 12:309. doi: 10.1038/s41398-022-02080-z, PMID: 35922402 PMC9349249

[50] Fervaha G Takeuchi H Lee J Foussias G Fletcher PJ Agid O . Antipsychotics and amotivation. Neuropsychopharmacology. (2015) 40:1539–48. doi: 10.1038/npp.2015.3, PMID: 25567425 PMC4397414

[51] Symes AL Sourkes TL Youdim MB Gregoriadis G Birnbaum H . Decreased monoamine oxidase activity in liver of iron-deficient rats. Can J Biochem. (1969) 47:999–1002. doi: 10.1139/o69-160, PMID: 5353933

[52] Pivina L Semenova Y Doşa MD Dauletyarova M Bjørklund G . Iron deficiency, cognitive functions, and neurobehavioral disorders in children. J Mol Neurosci. (2019) 68:1–10. doi: 10.1007/s12031-019-01276-1, PMID: 30778834

